# Comparison of the efficacy and safety between palliative biliary stent placement and duct clearance among elderly patients with choledocholithiasis: a propensity score-matched analysis

**DOI:** 10.1186/s12876-021-01956-6

**Published:** 2021-10-10

**Authors:** Koh Kitagawa, Akira Mitoro, Takahiro Ozutsumi, Masanori Furukawa, Yukihisa Fujinaga, Norihisa Nishimura, Yasuhiko Sawada, Tadashi Namisaki, Takemi Akahane, Hitoshi Yoshiji

**Affiliations:** 1 Department of Gastroenterology, Nara Medical University, 840 Shijo-cho, Kashihara, Nara, 634-8522 Japan; 2 Division of Endoscopy, Nara Medical University, Nara, Japan

**Keywords:** Aged, Choledocholithiasis, Endoscopic retrograde cholangiopancreatography, Lithotripsy, Stents

## Abstract

**Objectives:**

This study aimed to evaluate and compare the outcomes of palliative endoscopic biliary stenting (EBS) and complete stone removal among elderly patients with choledocholithiasis using propensity score matching.

**Methods:**

From April 2012 to October 2017, 161 patients aged 75 years and older with choledocholithiasis underwent endoscopic retrograde cholangiopancreatography at our institution. Among them, 136 (84.5%) had complete stone removal, and 25 (15.5%) underwent palliative EBS without further intervention until symptom occurrence. The median age of the EBS group was significantly higher than that of the complete stone removal group. The proportion of patients with dementia, cerebral infarction, preserved gallbladder with gallstones, and surgically altered anatomy was higher in the EBS group than in the complete stone removal group. Propensity score matching was used to adjust for different factors. In total, 50 matched patients (n = 25 in each group) were analyzed.

**Results:**

The median duration of cholangitis-free periods was significantly shorter in the EBS group (596 days) than in the complete stone removal group. About half of patients in the EBS group required retreatment and rehospitalization for cholangitis during the observation period. Cholangitis was mainly caused by stent migration. There was no significant difference in terms of mortality rate and procedure-related adverse events between the two groups. Death was commonly attributed to underlying diseases. However, one patient in the EBS group died due to severe cholangitis.

**Conclusions:**

Palliative EBS should be indicated only to patients with choledocholithiasis who have a poor prognosis.

## Introduction

Choledocholithiasis commonly occurs during the migration of gallstones from the gallbladder to the biliary tree. Gallstones are caused by the lower contractility of the biliary epithelium due to multiple factors including supersaturation of cholesterol in the bile, inadequate bile salt concentration and function, diet, hormone levels, and genetic predisposition [1, 2]. Furthermore, the incidence of choledocholithiasis increases with age [3]. Minimally invasive endoscopic procedures play an important role in the treatment of choledocholithiasis. Stone removal via endoscopic retrograde cholangiopancreatography (ERCP) is the first choice for the management of choledocholithiasis in recent years [4]. However, in some cases, complete stone removal can be challenging to perform due to patient characteristics such as age and underlying diseases, surgically altered anatomy, and stone factors including size and number [5]. Further, additional surgical cholecystectomy cannot be performed in elderly patients with poor general conditions who have undergone complete stone removal from the bile duct via ERCP. Thus, stone recurrence may occur [6–8]. In recent years, due to the aging population, palliative endoscopic biliary stenting (EBS) is sometimes the treatment of choice for high-risk elderly patients. EBS alone is associated with good survival among elderly patients who have not undergone complete stone removal [9–11]. Furthermore, some studies have shown that EBS can reduce the number and size of stones and facilitate stone removal for a period of time [12–15]. However, it is associated with stent-related complications such as stent occlusion and migration, and the American Society of Gastrointestinal Endoscopy (ASGE) guidelines state that management should aim at periodic stent replacement and eventual stent removal, without recommendations for permanent EBS [4]. Moreover, the efficacy of palliative EBS among frail elderly patients with choledocholithiasis remains controversial.

Large, high-quality studies conducted in the 1990s have reported the outcomes of palliative EBS [16, 17]. Since then, the aging of society has advanced. Furthermore, treatment techniques available for “difficult stones” are rapidly improving, and multiple large stones and surgically altered anatomy can now be managed endoscopically. However, in these previous studies, many younger patients were included, and cases of surgically altered anatomy were not included. Therefore, the efficacy of palliative EBS and duct clearance in elderly patients should be assessed and compared again at present. Therefore, this study aimed to retrospectively evaluate and compare the outcomes of palliative EBS and complete stone removal among elderly patients with choledocholithiasis using propensity score matching (PSM).

## Methods

### Study population

**Fig. 1:**
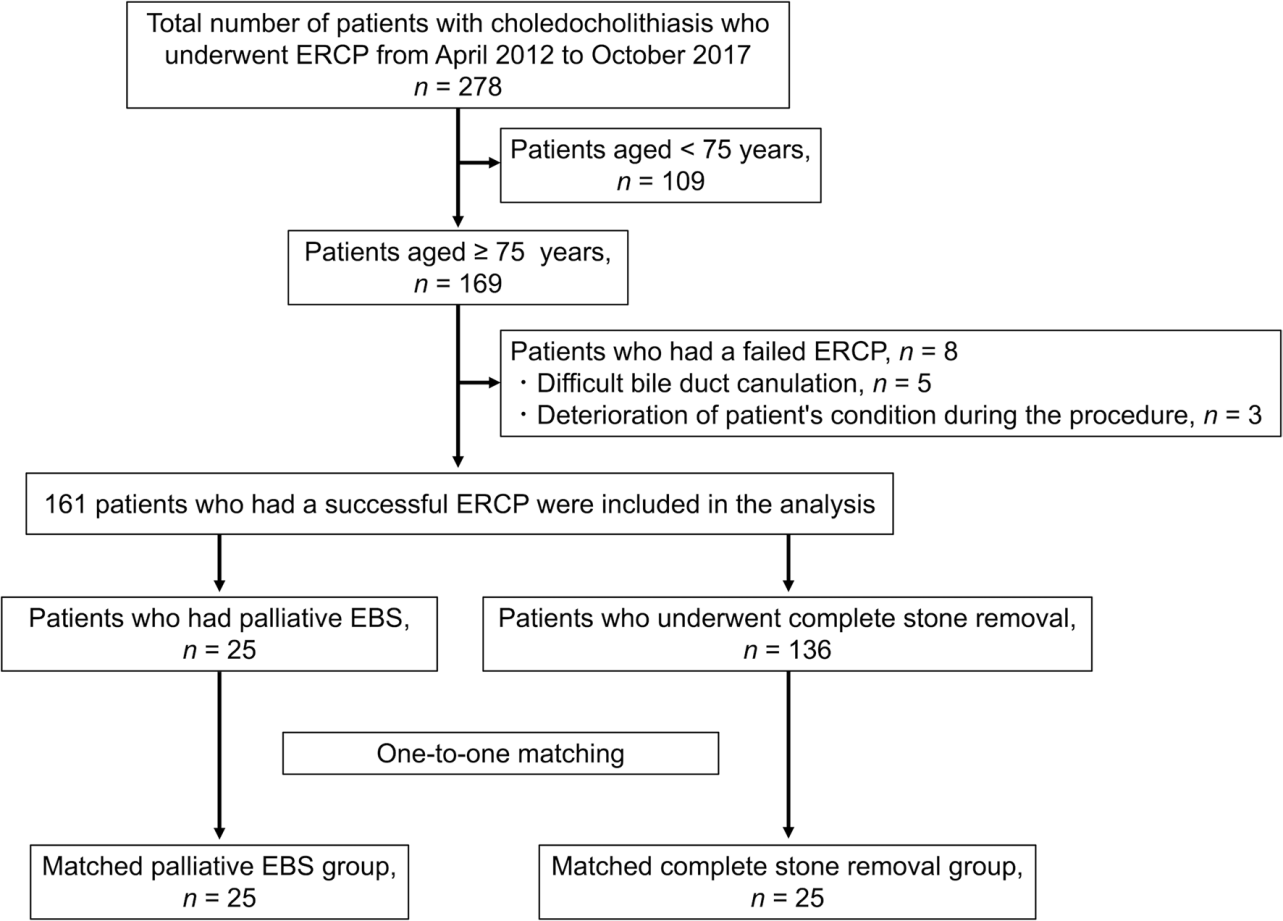
Flowchart of patient selection. Flowchart of patient selection into matched palliative EBS and complete stone removal groups (ERCP, endoscopic retrograde cholangiopancreatography; EBS, endoscopic biliary stenting)

The present study was approved by the institutional review board of Nara Medical University Ethics Committee (#1360). Figure 1 shows the patient flow chart in this study. From April 2012 to October 2017, 278 patients with primary choledocholithiasis underwent ERCP treatment at our institution. Among them, 109 who were aged younger than 75 years and 8 who had an unsuccessful ERCP were excluded from the study. Meanwhile, the remaining 161 patients were included in the analysis. In total, 136 (84.5%) patients had complete stone removal, and 25 (15.5%) underwent palliative EBS without further intervention until symptom occurrence. Patients with recurrence of choledocholithiasis following stone removal were not included in this study. The physician performed either complete stone removal or palliative EBS based on the patient’s condition and stone status. The EBS group also included cases in which stone removal was initially attempted but was difficult, and thereby palliative stenting was unavoidable with the stone remaining. None of the patients had routine or planned stent replacement. Patients who could visit our hospital were followed up every few months with blood tests and imaging examinations. Information regarding patient characteristics and endoscopic procedures was obtained from the medical records. We contacted the patients whose hospital visits were interrupted early and the families of such patients by phone, in addition to contacting their medical institutions to conduct as much prognostic research as possible. All authors had access to the study data and approved the final manuscript. This single-center retrospective study was performed in accordance with the Strengthening the Reporting of Observational Studies in Epidemiology (STROBE) Statement. Owing to the retrospective nature of this study, an opt-out approach was used instead of the requirement of written informed consent for participation in the study.

### ERCP

Patients were placed in prone position and were sedated with midazolam and buprenorphine hydrochloride, or haloperidol as appropriate. Vital signs were monitored with electrocardiogram and oxygen saturation assessment during ERCP. Patients received oxygen therapy via nasal cannula if needed. ERCP was performed using standard duodenoscopes (TJF260V and JF260V; Olympus Medical, Tokyo, Japan). A balloon-assisted endoscope (EC-450BL5; Fujifilm Medical, Tokyo, Japan) was used in all patients with surgically altered anatomy (except for the Billroth I method). Selective bile duct cannulation was performed with wire-guided cannulation and contrast medium injection methods using a standard ERCP catheter (MTW ERCP catheter; Medi-Globe GmbH, Rohrdorf, Germany) or a sphincterotome (CleverCut 3 V; Olympus Medical, Tokyo, Japan). EBS was performed using straight or pigtail plastic stents. In patients who underwent endoscopic papillary balloon dilation (EPBD), a 6- to 10-mm balloon catheter (HurricaneTM RX; Boston Scientific Japan, Tokyo, Japan) was used for balloon dilation. Endoscopic large balloon dilation (EPLBD) was performed using a 12- to 20-mm balloon catheter (CRE wire-guided balloon dilator; Boston Scientific Japan, Tokyo, Japan). In this study, none of the patients received rectal indomethacin during the periprocedural period.

### PSM

Table 1 shows the characteristics of patients. Although our study population was older than 75 years, a significant difference was noted in median age between the EBS group and the stone removal group in the total cohort before PSM. The EBS group had a significantly higher proportion of patients with dementia, history of brain stroke, preserved gallbladder with gallstones, surgically altered anatomy, periampullary diverticulum, and large bile duct and stones. However, there was no significant difference in stone number between the EBS and complete stone removal groups. In contrast, no significant difference was observed in stone number between the EBS and complete stone removal groups. No case of ERCP was observed in any of the patients with WHO performance status 4 in the either of the groups.

**Table 1:** Baseline characteristics of the total and propensity-matched cohorts for palliative EBS and stone removal

	Total cohort			Matched cohort		
	EBS (n = 25)	Complete stone removal (n = 136)	*p* value	EBS (n = 25)	Complete stone removal (n = 25)	*p* value
Age, median (range), years	86.0 (75–95)	81.0 (75–101)	< 0.01	86.0 (75–95)	84.0 (75–87)	0.20
Sex, male/female	14/11	85/51	0.66	14/11	17/8	0.56
WHO performance status score of 3, *n* (%)	2 (8.0)	9 (6.6)	0.68	2 (8.0)	2 (8.0)	1.00
Antiplatelet or anticoagulant therapy, *n* (%)	10 (40.0)	46 (33.8)	0.65	10 (40.0)	10 (40.0)	1.00
Cardiovascular disease, *n* (%)	9 (36.0)	36 (26.5)	0.34	9 (36.0)	10 (40.0)	1.00
Dementia, *n* (%)	10 (40.0)	16 (11.8)	< 0.01	10 (40.0)	8 (32.0)	0.77
History of cerebral infarction, *n* (%)	6 (24.0)	12 (8.8)	0.04	6 (24.0)	5 (20.0)	1.00
Malignant neoplasm, *n* (%)	6 (24.0)	28 (20.6)	0.79	6 (24.0)	7 (28.0)	1.00
*Gallstone*, *n* (%)						
Preserved gallbladder with gallstones	14 (56.0)	32 (23.5)	< 0.01	14 (56.0)	14 (56.0)	1.00
Periampullary diverticulum, *n* (%)	13 (52.0)	34 (25.2)	0.02	13 (52.0)	12 (48.0)	1.00
Surgically-altered anatomy^a^, *n* (%)	7 (28.0)	12 (8.8)	0.01	7 (28.0)	7 (28.0)	1.00
Diameter of common bile duct, median (range), mm	13.0 (7–23)	11.0 (4–28)	0.04	13.0 (7–23)	13.0 (6–28)	0.98
Diameter of stones, median (range), mm	10.0 (4–18)	8.0 (1–28)	0.02	10.0 (4–18)	10.0 (2–28)	0.93
Number of stones, median (range)	2.0 (1–10)	2.5 (1–20)	0.79	2.0 (1–10)	3.0 (1–10)	0.75

^a^Cases of Billroth-I reconstruction method were not included

PSM was performed to control for factors affecting the choice between complete stone removal and bile duct stenting. Two groups were matched at a 1:1 ratio (stone removal, n = 25; palliative EBS, n = 25) via a propensity score-matched analysis adjusted for 14 covariates (age, sex, WHO performance status, antiplatelet or anticoagulant therapy, cardiovascular disease, dementia, history of cerebral infarction, malignant neoplasm, gallstone status, periampullary diverticulum, surgically altered anatomy, diameter of the common bile duct, diameter of stones, and number of stones) for minimizing inherent bias. Each patient in the EBS group was matched to a patient in the stone removal group using the nearest-neighbor method with a caliper range of 0.20 of the standard deviation of the pooled propensity scores. All patient factors were eventually matched, and there was no significant difference between the two groups.

### Statistical analysis

Categorical variables were compared using the chi-square test or the Fisher’s exact test, and continuous variables using the *t*-test or the Mann–Whitney U test. A p value of < 0.05 was considered statistically significant. The duration of cholangitis-free periods was assessed using the Kaplan–Meier method and was compared between the two groups with the log-rank test. The duration of cholangitis- and death-free periods was censored at the end of each patient’s follow-up period. All statistical analyses were performed using EZR (version 1.41; Saitama Medical Center, Jichi Medical University, Saitama, Japan; http://www.jichi.ac.jp/saitama-sct/SaitamaHP.files/statmedEN.html), which is a graphical user interface for R (The R Foundation for Statistical Computing, Vienna, Austria, version 4.0.3) [18].

### Endpoints and definitions

The primary endpoint of our study was the time to recurrence of cholangitis after complete stone removal and palliative EBS among elderly patients with choledocholithiasis. The secondary endpoints were causes and severity of cholangitis, incidence of adverse events (AEs), mortality rate, and causes of death. If cholangitis developed after ERCP, its diagnosis and severity were defined according to the Tokyo Guideline 2018 [19]. Early AEs were defined as AEs occurring within 14 days, and late AEs as AEs occurring after 14 days after the procedure. AE severity was graded according to the ASGE lexicon [20].

## Results

### Endoscopic procedure and AEs

Table 2 depicts the endoscopic procedures. Three (12.0%) patients in the EBS group received ampullary interventions. These three patients had initially undergone ampullary interventions in an attempt to remove the stone completely, but it was difficult and the procedure was changed to palliative stenting with the stone remaining. Meanwhile, all patients in the stone removal group, including 18 (72.0%), 1 (4.0%), and 6 (24.0%) who underwent endoscopic sphincterotomy (ES), EPBD alone, and EPLBD with ES, respectively, had ampullary interventions. In the EBS group, straight-type plastic and pigtail-type stents were used in 68.0% and 32.0% of patients, respectively. The most common stents used were 7 cm in length and 7 F in diameter. The number of ERCP sessions was slightly higher in the stone removal group than in the EBS group. The procedure time was significantly shorter in the EBS group than in the stone removal group. Table 2 shows data about AEs other than cholangitis. In the EBS group, early AEs included acute pancreatitis (n = 1), aspiration pneumonia (n = 1), and retroperitoneal perforation (n = 1). In addition, one patient presented with acute cholecystitis, which is considered a late AE. Two patients in the stone removal group had acute pancreatitis. The incidence of AEs did not significantly differ between the EBS and complete stone removal groups (16.0% and 8.0%, *p* = 0.67). There were no procedure-related deaths in both groups.

**Table 2:** Endoscopic procedures and adverse events in the matched cohort

Total number of patients	EBS	Complete stone removal	*p* value
	n = 25	n = 25	
*Ampullary intervention*			
ES, *n* (%)	2 (8.0)	18 (72.0)	
EPBD, *n* (%)		1 (4.0)	
EPLBD, *n* (%)	1 (4.0)		
EPLBD with ES, *n* (%)		6 (24.0)	
*Type of stent*			
Straight type, *n* (%)	17 (68.0)		
Pig tail type, *n* (%)	8 (32.0)		
*Length of stent*			
5 cm/7 cm/9 cm/10 cm, *n*	9/13/2/1		
*Diameter of stent*			
7Fr/8.5Fr, *n*	22/3		
*Total number of ERCP sessions*			
1, *n* (%)	22 (88.0)	17 (68.0)	0.17
2–3, *n* (%)	3 (12.0)	8 (32.0)	
*Procedure time, median (range), minutes*	17 (5–181)	41 (10–88)	< 0.01
*Adverse events*^a^ *(AE), n (%)*	4 (16.0)	2 (8.0)	0.67
Early AEs			
Acute pancreatitis (mild)	1 (4.0)	2 (8.0)	
Aspiration pneumonia (mild)	1 (4.0)		
Retroperitoneal perforation (severe)	1 (4.0)		
Late AEs			
Acute cholecystitis (moderate)	1 (4.0)		

*ES* endoscopic sphincterotomy, *EPBD* endoscopic papillary balloon dilatation, *EPLBD* endoscopic large balloon dilatation

^a^Except for cholangitis or stent-related adverse events

### Duration of cholangitis-free periods

**Fig. 2:**
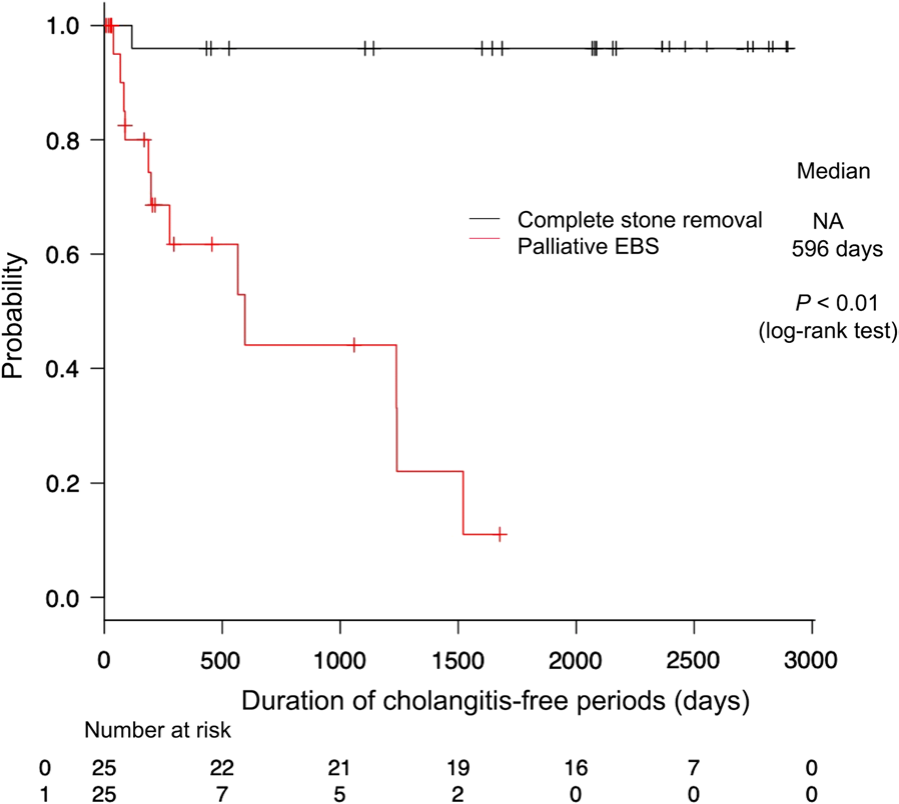
Duration of cholangitis-free periods. Kaplan–Meier graph showing the duration of cholangitis-free periods. The duration of EBS was significantly shorter than that of complete stone removal (*p* < 0.01, log-rank test; EBS, endoscopic biliary stenting; NA, not applicable)

The Kaplan–Meier curve for cholangitis-free duration is shown in Fig. 2. The median cholangitis-free period in the EBS group was 596 days (95% confidence interval, 187–1240 days); however, the median cholangitis-free period in the stone removal group could not be estimated during the observation period. The stone removal group had significantly longer cholangitis-free period than the EBS group (Log-Rank test; *p* < 0.01). Table 3 shows the incidence, cause, and severity of cholangitis. About half of patients in the EBS group developed cholangitis, and it was significantly more common in the EBS group than in the stone removal group (*p* < 0.01). Stent migration was the most common cause of cholangitis in the EBS group. We also investigated the correlation between stent geometry and migration (Table 4) and found no significant difference in the frequency of stent migration between the pigtail and straight types of stents.

**Table 3:** Cholangitis and mortality

Total number of patients	EBS	Complete stone removal	*p* value
	n = 25	n = 25	
Follow-up period, median (range), days	456 (8–1676)	378 (28–2364)	0.88
*Cholangitis, n (%)*	12 (48.0)	1 (4.0)	< 0.01
Causes of cholangitis			
Stent migration, *n* (%)	7 (28.0)		
Stent occlusion^a^, *n* (%)	5 (20.0)		
Recurrence of choledocholithiasis, *n* (%)		1 (4.0)	
Severity of cholangitis			
Moderate, *n* (%)	11 (44.0)	1 (4.0)	
Severe, *n* (%)	1 (4.0)		
*Mortality, n (%)*	3 (12.0)	2 (8.0)	1.00
Causes of mortality			
Pneumonia	1 (4.0)		
Renal failure	1 (4.0)		
Severe cholangitis	1 (4.0)		
Gastric cancer		2 (8.0)	
Length of hospital stay, median (range), days	16 (2–396)	11 (2–35)	0.27

^a^Including a patient with stent–stone complex formation

**Table 4:** Correlation between stent type and migration

	Straight type	Pigtail type	*p* value
	n = 18	n = 7	
Stent migration, *n* (%)	4 (22.2%)	3 (42.9%)	0.64

**Fig. 3:**
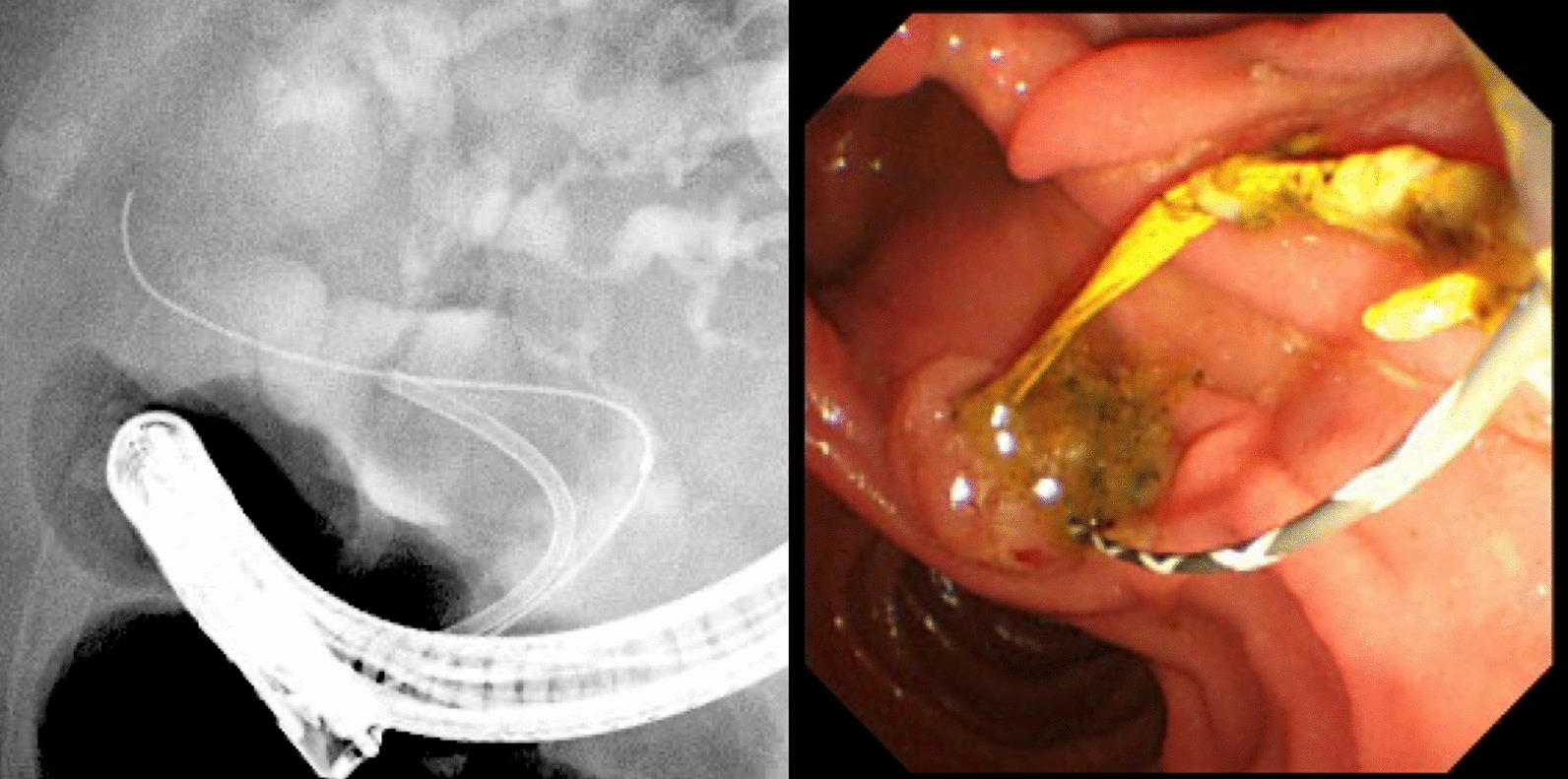
Stent–stone complex. One patient in the palliative EBS group had stent–stone complex. He developed cholangitis 3 years after the initial ERCP stent placement. ERCP was performed again, and the stent ruptured upon removal (EBS, endoscopic biliary stenting; ERCP, endoscopic retrograde cholangiopancreatography)

Furthermore, one patient in the EBS group had stent–stone complex. Thus, the old stent was difficult to remove (Fig. 3). By contrast, only one patient in the complete stone removal group presented with cholangitis, which was caused by recurrence of bile duct stones. All patients with cholangitis were treated again with ERCP, and all reinterventions were technically successful. Most patients presented with moderate cholangitis. However, one patient in the EBS group died due to severe cholangitis.

### Mortality

During a median observation period of 442.5 (range: 8–2364) days, 3 (12.0%) patients in the EBS group and 2 (8.0%) in the complete stone removal group died. There was no significant difference in terms of mortality rate between the two groups. Death was commonly caused by underlying diseases. However, one patient in the EBS group died due to severe cholangitis. The length of hospital stay was also examined, and the result did not significantly differ between the two groups (Table 3).

## Discussion

Endoscopic lithotripsy is the first choice for the treatment of choledocholithiasis. Minimally invasive endoscopic treatment, which is safe even among frail elderly patients with underlying diseases, has been increasingly used. However, in actual clinical practice, patients may not endure long procedures. Thus, the procedure can be discontinued, thereby leaving a biliary stent alone. Bergman et al. assessed the outcomes of long-term treatment with 10-F polyethylene stents among elderly patients (n = 58) [16]. This method was initially successful. However, over time, 38% of patients developed recurrent cholangitis; in 12% of cases, it was fatal. Chopra et al. found that ductal clearance was consistently associated with a higher rate of procedural AEs in a randomized comparison between ductal clearance and long-term biliary stenting (16% vs. 7%). However, the incidence of long-term biliary AEs was lower (14% vs. 36%) [17]. By contrast, there are some reports showing that long-term stenting is beneficial for high-risk elderly patients with choledocholithiasis [9–11]. However, the validity of long-term EBS remains controversial.

In retrospective studies comparing complete stone removal and long-term EBS, selection bias is more likely to occur because a high number of elderly patients and those with poor general health are included in the EBS group. In this study, this bias was eliminated by including only patients aged 75 years and older in the target population and by matching patient factors between the two groups via PSM.

Interestingly, when we compared the background characteristics of patients before PSM in our study, we found significant differences in terms of patient factors (such as age and underlying diseases) and anatomical factors (including periampullary diverticulum and surgically altered anatomy). However, there was no significant difference in terms of the number of stones. Furthermore, the patients in the EBS group were more likely to have larger stones. Nevertheless, the median diameter was only 10 mm. In recent years, the technique used to remove the so-called difficult stones has significantly improved. New ampullary interventions, including EPLBD, were found to be useful in the treatment of large and multiple stones [21–23]. Moreover, lithotripsy techniques including electronic hydraulic lithotripsy using a digital-single-operator cholangioscopy have also been developed [24, 25]. This suggests that the general condition of the patients, rather than stone factors, affects the choice of palliative EBS. In addition, in the total cohort before PSM, the EBS group had a significantly higher proportion with surgically altered anatomy. In recent years, ERCP with a balloon-assisted endoscope has been found to be effective in patients with surgically altered anatomy [26–28]. However, the technique is still challenging. This might increase the number of cases in which the stones are not completely removed, even after a successful ERCP, and the procedure was discontinued with stenting performed alone.

Indeed, there were some cases in the EBS group in which the procedure required an extensive amount of time, even for stenting alone, caused by the difficulty of scope insertion in patients with surgically altered anatomy. As shown in Table 2, the procedure time in the palliative EBS group varied (range 5–181 min). No significant difference was noted in the incidence of procedure-related AEs between the two groups, but the incidence of AEs tended to be slightly higher in the EBS group. This finding may be associated with the fact that there were some high-risk patients in the EBS group who underwent prolonged procedures, as described above, or who were initially planned to undergo stone removal that was discontinued midway through the procedure and instead underwent palliative stenting. There were no procedure-related deaths in either of the groups. In contrast, the median time to cholangitis was significantly shorter in the EBS group than in the complete stone removal group. There was no significant difference between the two groups in terms of treatment safety or length of hospital stay. Nevertheless, about half of the patients in the EBS group eventually required reintervention and rehospitalization. More importantly, one patient in the EBS group died due to severe cholangitis. In the EBS group, the main cause of stent dysfunction was stent migration, which might have been caused by the fact that the stent was placed in the bile duct without stenosis. It was difficult to control migration, even with pigtail-type stents. Additionally, in our study, there was a case of stent–stone complex in the EBS group. Kaneko et al. showed that long-term EBS increases the risk of stent–stone complex [29]. Stent–stone complex formation can lead to difficulties in removing old stents via conventional endoscopic procedures. Therefore, palliative EBS may be acceptable in patients with malignancies who have a poor prognosis. However, the indications should be limited. Even in cases in which biliary stenting was unavoidable at the time of initial treatment, it may be necessary to perform the procedure again, after the patient’s general condition improves, to achieve complete stone removal or to consider planned stent replacement [30].

By contrast, the present study showed a relatively long duration of cholangitis-free period in the EBS group (596 days). By employing a transpapillary stent placement approach in the bile duct, even if the stent was occluded, the impaction of the stones on the duodenal papilla could have been inhibited and incidence of cholangitis may have been less likely. This study also included some cases of patients with surgically altered anatomy; incidence of stent occlusion was possibly less likely in these cases because food residue did not pass through the afferent limb.

In addition, in some elderly patients with choledocholithiasis, even if the bile duct stones are removed via ERCP, the gallbladder stones may not be surgically resected thereafter. In our study, even in the matched cohort adjusted for the proportion of patients with residual gallbladder with gallstones after ERCP, the incidence of cholangitis was significantly lower in the stone removal group. Yasui et al. showed that the recurrence of choledocholithiasis did not increase even if the gallbladder with gallstones is preserved after endoscopic treatment of choledocholithiasis among elderly patients [31]. Therefore, regardless of whether cholecystectomy is feasible in the future, a reasonable therapeutic strategy should be used to completely remove bile duct stones.

The current study had several limitations. First, patients with inadequate follow-up were included. This study included several frail and elderly patients with underlying medical conditions. In some cases, regular outpatient visits were challenging. Therefore, long-term prognosis could not be assessed in such cases. We attempted to conduct as much prognostic research as possible through follow-up of the patients using telephone surveys and inquiries to their medical institutions, however, we may have failed to detect mild cases of cholecystitis and cholangitis. In our study, the incidence of cholangitis and cholecystitis was lower in the stone removal group, and the duration of the cholangitis-free period was relatively longer in the EBS group. However, we cannot deny the possibility that the incidence of cholecystitis and cholangitis noted in both groups may have been lower than the actual incidence. Second, this is a retrospective study, which may not be as statistically reliable as randomized control trials. However, conducting a prospective randomized controlled trial comparing EBS and stone removal in frail and elderly patients is not easy. In this study, the background characteristics of the stone removal group and the EBS group were adjusted using PSM to ensure homogeneity between the two groups. We believe that the statistical reliability of this study is sufficient. Third, because the outcome of the study was the occurrence of cholangitis, the exact recurrence rate of choledocholithiasis in the stone removal group remains unknown. However, we believe that the incidence of cholangitis is more important than the recurrence rate of stones in clinical practice. Therefore, this study is considered more relevant to actual clinical practice.

In conclusion, palliative EBS was effective in controlling cholangitis for a certain period of time among frail elderly patients with choledocholithiasis. However, a significantly higher number of patients required reintervention and rehospitalization for cholangitis in the EBS group than in the complete stone removal group. The median duration of cholangitis-free periods in the palliative EBS group was significantly shorter than that in the complete stone removal group even after adjusting for background characteristics using PSM. Furthermore, one patient in the EBS group died due to severe cholangitis. Thus, palliative EBS should be indicated only in patients with choledocholithiasis who have a poor prognosis.

## Data Availability

The datasets used and/or analyzed during the current study are available from the corresponding author on reasonable request.
